# CAR-T cell therapy: a potential new strategy against prostate cancer

**DOI:** 10.1186/s40425-019-0741-7

**Published:** 2019-10-16

**Authors:** Giuseppe Schepisi, Maria Concetta Cursano, Chiara Casadei, Cecilia Menna, Amelia Altavilla, Cristian Lolli, Claudio Cerchione, Giovanni Paganelli, Daniele Santini, Giuseppe Tonini, Giovanni Martinelli, Ugo De Giorgi

**Affiliations:** 1 Department of Medical Oncology, Istituto Scientifico Romagnolo per lo Studio e la Cura dei Tumori (IRST) IRCCS, Via P. Maroncelli 40, 47014 Meldola, Italy; 2 Campus Bio-Medico University, Rome, Italy

**Keywords:** T cells, Prostate cancer, CAR-T, Immunotherapy

## Abstract

Prostate cancer (PCa) is one of the main causes of cancer-related death in men. In the present *immunotherapy era*, several immunotherapeutic agents have been evaluated in PCa with poor results, possibly due to its low mutational burden. The recent development of chimeric antigen receptor (CAR)-T cell therapy redirected against cancer-specific antigens would seem to provide the means for bypassing immune tolerance mechanisms. CAR-T cell therapy has proven effective in eradicating hematologic malignancies and the challenge now is to obtain the same degree of in solid tumors, including PCa. In this study we review the principles that have guided the engineering of CAR-T cells and the specific prostatic antigens identified as possible targets for immunological and non-immunological therapies. We also provide a state-of-the-art overview of CAR-T cell therapy in PCa, defining the key obstacles to its development and underlining the mechanisms used to overcome these barriers. At present, although there are still many unanswered questions regarding CAR-T cell therapy, there is no doubt that it has the potential to become an important treatment option for urological malignancies.

## Introduction

Prostate cancer (PCa) remains one of the main causes of cancer-related death in men. Although it is often a manageable tumor, around 20% of patients develop metastases and the disease eventually evolves into metastatic castration-resistant PCa (mCRPC) [1]. In the last few years, new drugs have been evaluated for the treatment of mCRPC and, following Food and Drug Administration (FDA) approval of sipuleucel-T (Dendreon Corporation), several studies have been conducted to assess the role of immunotherapeutic agents, including new checkpoint inhibitors, in this setting [2, 3]. No immune checkpoint inhibitor (as monotherapy) has demonstrated efficacy in PCa thus far [4–6]. In particular, no overall survival (OS) benefit has been observed in patients treated with ipilimumab [7–9], whereas monotherapies directed against PD1 or PD-L1 have only demonstrated limited response in PCa patients, probably due to an immunologically cold PCa microenvironment [10]. Moreover, the role of PD-L1 status in PCa patients is controversial. Recently, Li et al. demonstrated its expression as a negative independent prognostic factor in PCa patients. PD-L1 overexpression has also been correlated with high Gleason scores and androgene receptor positivity [11]. PD-L1 overexpression appears to be higher in metastatic sites than in primary PCa [12], especially in enzalutamide-pretreated patients [13]. Interestingly, Calagua et al. did not find any difference in PD-L1 expression between treated and untreated mPCa patients [14]. At ASCO GU 2019, results from the CheckMate 650 phase II trial (NCT02985957) revealed the efficacy of the combination of the CTLA4-inhibitor ipilimumab and the PD-1-inhibitor nivolumab. In a cohort of mCRPC patients pretreated with taxane and hormone therapy, 10% (3/30) showed a response at a median follow-up of 13.5 months, while in the other cohort pretreated with 2 hormone therapy lines, 25% (8/32) had a response at a median follow-up of 11.9 months [15].

In this scenario, the development of genetically engineered T cells capable of overcoming cancer immunological tolerance would represent an important step forward in cancer research. In the current ‘*new era’* of cancer immunotherapy, clinical trials have been carried out to verify the potential for using chimeric antigen receptor (CAR) T cells to identify and eliminate malignant cells. CAR-T is a molecule consisting of a tumor antigen-binding domain fused to an intracellular signaling domain and costimulatory molecules [16]. For this reason, antigen-identification is not major histocompatibility complex (MHC)-restricted, as is the case of T cell receptor (TCR)-mediated antigen recognition.

The first studies were conducted on hematological tumors and showed high response rates and durability of remission in chronic lymphocytic leukemia (CLL) acute lymphoblastic leukemia (ALL), and refractory B cell lymphoma [17–23]. Such excellent results led to FDA approval of CD19-directed CAR-T cells for the treatment of relapsed/refractory pediatric and young-adult diffuse large B cell lymphoma (DLBCL), also sparking off research into solid tumors. The characteristic of being monoclonal diseases and the consequent identification of the same target antigen for all neoplastic cells is probably the main reason for the success of CAR-T cell therapy in hematological malignancies. In solid tumors, polyclonality, physical barriers and tumor microenvironment probably account for the difficulties in obtaining the same promising results. However, the recent identification of specific PCa membrane antigens can be considered the starting point that has led to the development of cell-directed immunotherapy.

In this review we provide an in-depth overview of CAR-T cell therapy in PCa and suggest strategies to further improve current results.

## CAR-T structure

PCa is associated with a low mutational burden. CAR-T cells are synthetic molecules in which the effector function of T lymphocytes combines with the ability of antibodies to identify specific antigens. Thus, CAR T cells do not require antigen presentation by antigen presenting cells (APC) and can recognize intact proteins. Consequently, the creation of genetically engineered T cells redirected to tumor antigens bypasses several mechanisms of immunological tolerance [24]. Recent studies have shown that the “optimal” T cell population for the generation of CAR-T cells are poorly differentiated cells, i.e. the earliest memory T cells (stem cells memory T). The modifications occurring during T cell maturation process (in particular, loss of co- stimulatory receptors and erosion of telomeres) make differentiated T cells less suitable [25–27].

CAR molecules can be divided into 3 components: 1) an extracellular domain, which is involved in antigen identification. This zone is composed of a single-chain fragment variable (scFv) that (specifically) recognizes tumor-associated antigens (TAA). scFV is fixed on T cell by a 2) transmembrane domain, composed of a transmembrane region of CD3, CD8, CD28 or FcεRI. This region is connected to the 3) intracellular zone which is composed of the intracytoplasmic region of CD8, CD28 or CD137 and CD3ζ. This last zone comprises the immune receptor tyrosine-based activation motif (ITAM) which, in turn, plays a fundamental role in signal transduction aimed at activating T cells [28].

To date, in vitro transfection technology is the standard method to transfect CAR molecules into T lymphocytes. Transfection can be achieved through viral (retro- or slow virus) or non-viral (transposon and mRNA electrotransfection) methods.

**Fig. 1:**
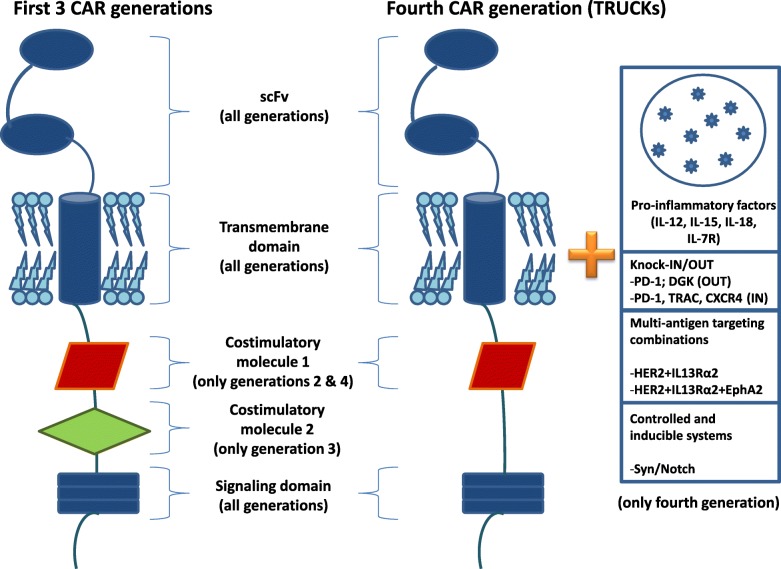
Different characteristics of chimeric antigen receptor (CAR) generations. scFv, single-chain fragment variable

Generally, CARs are classified into 4 types based on molecular complexity (Fig. 1): the first type comprises CARs with only a simple receptor divided into the above-mentioned 3 components (scFv, transmembrane domain and intracellular zone). These CAR-T constructs permit T cell activation but, given the lack of a costimulatory molecule, this first generation failed to obtain significant results in terms of persistence of T-lymphocyte activation in blood circulation [29–31]. To overcome this problem, a second CAR generation was developed by inserting the intracellular domain of a costimulatory protein, such as CD28, CD27, CD134 or CDB7. Another costimulatory molecule (CD28, 4-1BB, or CD3ζ) was added to develop a third CAR generation aimed at increasing the extent of T-cell activation [32]. The fourth generation of these molecules (also known as TRUCK, i.e. T cells redirected for universal cytokine-mediated killing, or CAR-T cells armed with immune stimulatory cytokine) has both a costimulatory element and proinflammatory factor, such as interleukin (IL)-12, which increases T-cell efficacy [33]. In fact, the presence of IL-12 counterbalances the immunosuppressive action of the tumor microenvironment by inducing a shift in the T-cell response towards a T helper-1 type [34, 35]. However, the fourth generation of CAR is not limited to IL-12 alone, different types of molecules having been developed for use in the construction of TRUCKs. These include cytokines such as IL-15 (similar to IL-12, this interleukin enhances the development of T-memory stem cells) [36] and IL-18 [37], and also constitutively active cytokine receptors such as IL-7 receptor (C7R) whose aim is to overcome the risk of cytokine toxicity [38]. Other molecules tested in TRUCKs are knock-out genes (PD-1 or DGK) and knock-in genes (TRAC or CXCR4), their aim to improve CAR expression and anti-tumor activity [39, 40]. Controlled and inducible systems (Syn/Notch) and multiantigen combinations (HER2 + IL13Rα2) have also been used to prevent antigen escape [41].

## Prostate TAAs and known immunotherapy strategies

The identification of prostate TAAs is the first step towards developing an effective CAR-T cell therapy. An ideal antigen should be constitutive and specifically expressed by cancer cells to enable CAR-T cells to develop a cancer-specific immunologic response, thus sparing healthy tissue [42, 43]. In PCa the group of protein preferentially expressed by malignant cells are prostate-specific antigen (PSA), prostatic acid phosphatase (PAP), prostate stem cell antigen (PSCA), T-cell receptor gamma alternate reading frame protein (TARP), transient receptor potential (trp)-p8 and prostate-specific membrane antigen (PSMA). In recent years, several studies have used prostate TAAs as a target for the induction of an immunological response in PCa patients [44, 45] (Table 1).

**Table 1:** Pros and Cons of using each TAA in the development of CAR-T cells in prostate cancer

TAA	Description	Pros	Cons
PSA	Serine protease	1) Almost exclusively expressed by prostate epithelial cells 2) Stimulates cytotoxic lymphocytes in vivo [45]	It is a prostate-specific but not tumor-specific antigen
PAP	Tyrosine phosphatase protein	1) Secreted by benign and malignant prostate cells 2) Stimulates CTLs in vivo [45]	1) More highly expressed in Gleason score 6 and 7 tumors than in higher Gleason score tumors. 2) Expressed in the placenta, kidneys and testes, and also in gastric, breast and colon cancer
PSCA	Serine protease	Expression increases with both high Gleason score and metastasis	PSCA has also been found expressed in other cancer types
PSMA	Transmembrane protein	Enhances cytokine production	Also expressed in low levels in salivary glands, brain and kidneys
EpCAM	Transmembrane protein	Show significance as a biomarker for early cancer development	Not a prostate cancer- specific antigen

*Abbreviations*: *TAA* tumor associated antigen, *PSA* prostate-specific antigen, *PAP* prostatic acid phosphatase, *PSCA* prostate stem cell antigen, *PSMA* prostate-specific membrane antigen, *EpCAM* epithelial cell adhesion molecule precursor

## PSA

Preclinical studies in transgenic mice have shown that PSA, a kallikrein-like serin-protease almost exclusively expressed by prostate epithelial cells, induces a specific T-cell response. Arredouani et al. generated a transgenic mouse expressing human PSA in the prostate and crossed it to the human leucocyte antigen (HLA-A2.1 transgenic mouse to assess whether androgen deprivation affects T-cell response, observing a significant increase in PSA-specific cytotoxic lymphocytes, especially after androgen ablation [46].

## PAP

PAP is secreted by benign and malignant prostate cells and is more highly expressed in Gleason score 6 and 7 tumors then in higher Gleason score tumors. It is not really a specific prostate antigen because it is expressed in the placenta, kidneys and testes, and also in gastric, breast and colon cancer. Kantoff et al. presented the results of a phase III trial that led to the FDA approval of sipuleucel-T for the treatment of asymptomatic or minimally symptomatic mCRPC. In the trial, PCa patients in the experimental arm were treated with APCs pre-exposed in vitro to PA2024, a fusion protein consisting of human granulocyte-macrophage colony-stimulating factor and PAP [47]. The sipuleucel-T patient group experienced a 22% relative reduction in risk of death compared to the placebo group, the reduction representing a 4.1-month improvement in median survival.

Patients enrolled in the experimental arm experienced chills, fever, and headache as adverse events.

## PSCA

PSCA is a cell surface glycoprotein expressed by prostate cells and carcinomas with a higher Gleason score. Several studies have evaluated the activity of in vitro*-*generated tumor-reactive CTL response by HLA-A2-restricted anti-prostate stem cell antigen (PSCA) peptides [48–50]. Other studies have been conducted on the TRAMP mouse model with PSCA-expressing PCa. Following vaccination with a viral vector encoding PSCA, TRAMP mice developed an antigen-specific CTL response that subsequently inhibited PCa progression [51, 52]. PSCA has also been evaluated as a target for antibody-based immunotherapy. Both conjugated and unconjugated anti-PSCA antibodies have shown activity against PCa cells, resulting in cytotoxicity and regression of xenografts in mice [53–55]. Taking into account the potential immunological effect of PSCA, Morgenroth et al. modified T cells by transducing chimeric antigen receptors that specifically recognize PSCA. The engineered T cells efficiently lysed PSCA-expressing cells [56].

## PSMA

PSMA is a transmembrane glycoprotein (also known as FOLH1) with relative specificity as a PCa cell-surface ligand [57]. Moreover, its expression progressively increases as higher grade tumors [58] and correlates with castration-resistant disease. Its role in positron emission tomography (PET) was confirmed by Caroli et al. in a prospective series of patients with biochemical recurrence of PCa, the authors reporting the superior performance and safety of ^68^Ga-PSMA PET/CT over choline PET/CT [59].

The potential of PSMA has been investigated in targeted therapy and in immunotherapy, some studies showing that HLA-A2-restricted PSMA-derived peptides induce antitumoral CTL responses in vitro [60–63]. Other studies in vitro and in xenograft models have evaluated PSMA as a target molecule for immunotherapy with conjugated and unconjugated antibodies directed against PSMA-expressing PCa cells [64–67]. Over the last decade, PSMA has been studied in vitro and in vivo to optimize antigenic stimulation of T-cell response through engineered T-cells expressing chimeric anti-PSMA immunoglobulin-T-cell-receptor constructs. In our Institute, PSMA conjugated with 177Lutetium (177Lu-PSMA) is being evaluated for safety and efficacy in an ongoing single-arm phase II trial of radiometabolic therapy for advanced castration-resistant PCa (NCT03454750). The phase III open label VISION Trial is currently recruiting 750 patients with progressive PSMA-positive PCa pretreated with abiraterone or enzalutamide and one or 2 lines of taxane-based chemotherapies. Patients are randomized to receive either 177Lu-PSMA-617 plus best supportive/best standard care or best supportive/best standard care alone, the aim being to compare overall survival (OS) between the two arms (NCT03511664). Other studies, not yet recruiting, have been designed to examine the safety, tolerability and efficacy of the combination of 177Lu-PSMA with pembrolizumab (NCT0365844) or olaparib (NCT03874884).

## Prostein, TARP, trp-p8

Prostein and trp-p8 are transmembrane proteins expressed in normal and malignant prostate tissue, while TARP is present in the mitochondria of PCa cells. Several preclinical trials have evaluated their efficacy in stimulating CTL response [68–71]. Recently, a pilot study of PSMA and TARP peptide vaccine with poly IC-LC (Hiltonol) as adjuvant was performed in HLA-A2 (+) hormone-naive PCa patients with elevated PSA after initial definitive treatment (NCT00694551). The aim of the study was to establish the safety and toxicity of varying doses of the vaccine and to assess its impact on PSA. The results are still incomplete but no serious adverse events have been recorded to date.

## CAR-T cells in metastatic PCa

Few studies evaluating CAR-T cell therapy in metastatic prostate cancer (mPCa) have been conducted to date (Table 2), PSMA and PSCA representing the most important candidates as CAR-T cell-targeted antigens.

**Table 2:** CAR-T cell therapy studies on prostate cancer

Publication	Publication year	Country	Institution	Setting	Cell source and type	Generation	Costimulatory domain
Gade et al [72]	2005	USA	Memorial Sloan-Kettering Cancer Center	Preclinical - mice model	Anti PSMA CAR-T cells	First generation	–
Maher et al [73]	2002	USA	Memorial Sloan-Kettering Cancer Center	Preclinical	Anti PSMA CAR-T cells	Second generation	CD28
Zuccolotto et al [74]	2014	Italy	University of Padua	Preclinical - mice model	Anti PSMA CAR-T cells	Second generation	CD28
Ma et al [75]	2014	USA	Roger Williams Med Center	Preclinical - mice model	Anti PSMA CAR-T cells	Second generation	CD28
Slovin et al [76]	2017	USA	Memorial Sloan-Kettering Cancer Center	Phase I NCT01140373	Autologous T Anti PSMA CAR-T cells	Second generation	CD28
Kloss et al [77]	2019	USA	University of Pennsylvania	Preclinical - mice model	Anti PSMA-TGFβ insensitive CAR-T cell	Second generation	4-1BB
Zhang et al [78]	2018	UK	Oxford University	Preclinical - mice model	Anti PSMA-TGFβ insensitive CAR-T cell	Second generation	4-1BB
Hassani et al [79]	2019	Iran	Tehran Univ Med Science	Preclinical - mice model	VHH-CAR-T cell anti PSMA	Second generation	CD28
Priceman et al [80]	2017	USA	City of Hope, Duarte	Preclinical - mice model	Anti PSCA CAR-T cell	Second generation	4-1BB
Hillerdal et al [81]	2014	Sweden	Uppsala University	Preclinical - mice model	Anti PSCA CAR-T cell	Third generation	CD28, OX-40 CD3ζ
Kloss et al [82]	2012	USA	Memorial Sloan-Kettering Cancer Center	Preclinical - mice model	Anti PSCA/Anti PSMA CAR-T cell	Third generation	4-1BB, CD28
Feldman et al [83]	2017	Germany	Institute of radiopharmaceutical Cancer Research, Dresden	Preclinical - mice model	Anti PSCA/Anti PSMA CAR-T cell	Second generation	CD28
Deng et al [84]	2015	China	Cancer Hospital & Institute	Preclinical - mice model	Anti EpCAM CAR-T cell	Second generation	CD28
NCT03873805	2019	USA	City of Hope, Duarte	Phase I	Anti PSCA CAR-T cell	Second generation	4-1BB

*Abbreviations*: *CAR* chimeric antigen receptor, *PSMA* prostate-specific membrane antigen, *TGFβ* transforming growth factor β, *PSCA* prostate stem cell antigen, *EpCAM* epithelial cell adhesion molecules, *VHH* camelid nanobody

### PSMA-CAR-T cells

In vitro and in vivo models have shown that PSMA-CAR-T cells proliferate and recognize PSMA+ cells [72, 73]. An in vivo study by Zuccolotto et al. on the activity of PSMA-CAR-T cells in mPCa revealed that these cells can survive in mice with diabetes/severe combined immunodeficiency. The treatment proved capable of eradicating mPCa in the preclinical setting [74].

Second-generation CAR-T cells show a better killing effect than those of the previous generation and represent a novel immune-targeted approach for mPCa [75]. Slovin et al. investigated an anti-PSMA CAR-T cell therapy in a phase I clinical trial of mPCa patients (NCT01140373). The authors assessed the safety of various doses and developed a protocol for ex-vivo transduction, expansion and clinical administration of the treatment [76]. Another phase I trial (NCT03089203) is currently testing the safety and feasibility of dual PSMA-specific/TGFβ-resistant, lentivirally transduced, CAR-modified autologous T cells (CART-PSMA-TGFβRDN cells) [77].

Ma et al. fabricated a second-generation anti-PSMA CAR-T cell therapy by inserting the co-stimulator CD28, testing it in mice [75]. Tumor volume decreased significantly (virtually disappearing after 3 weeks) in mice inoculated with anti-PSMA CAR-T cells with respect to those inoculated with non-transduced T cells. Zhang et al. recently developed a CAR-T cell therapy specific for PSMA and resistant to transforming growth factor β (TGF-β) by infecting CD8^+^ T cells from mCRPC patients with a retroviral construct. The construct carried an anti-PSMA chimeric T-cell receptor (TCR) gene and a dominant negative TGF-β type II gene, the former conferring T-cell specificity and the latter, resistance to TGF-β-mediated suppression of cytotoxic T lymphocytes. The engineered CAR-T cells had ganciclovir as a safety mechanism thanks to their expression of HSV1 thymidine kinase. The CAR-T cells increased 23.4-fold in 21 days and ganciclovir decreased survival to 1.5% in 5 days. In a mouse xenograft model, treatment with PSMA-specific and TGF-β-insensitive CAR-T cells led to lysis of PSMA-expressing PC3 tumors but not of normal PC3 tumors. Tumor apoptosis, CD8^+^ cell infiltration and increased interferon-gamma (IFNγ) and interleukin-2 (IL-2) levels were only seen in PSMA-positive PC3 tumors [78].

Hassani et al. recently constructed a CAR-T cell therapy against PSMA using camelid nanobody (VHH) [79]. For the first time scFvs of murine origin were not used in the CAR-T cell structure because of its limitations with regard to the immunogenicity of mouse antigens in humans and the relatively large size of scFvs. The specificity of VHH-CAR-T cells against PSMA^+^cells was confirmed by the increase observed in interleukin-2 (IL-2) cytokine and in CD69 expression (around 38%) [79].

### PSCA-CAR-T cells

With regard to PSCA, a first-generation CAR with scFv of 7F5 antibody led to the activation of an anti-tumor response in mice [80]. In a recent study, Priceman et al. evaluated the role of co-stimulation in PSCA-CAR-T cell activity. Comparing the co-stimulation activity of both CD28 and 4-1BB, the authors found that the latter molecule was more effective in activating T-cells than the former, thus paving the way for further analyses in this field [81].

A PSCA-CAR-T cell-mediated delay in tumor growth was obtained in mice using 1G8 and Ha1–4.117 antibodies [82], suggesting that CAR-T cell cytotoxicity may not be sufficient for in vivo treatment. A potential solution might be to develop a combined low-affinity PSCA-CAR-T and high-affinity PSMA-CAR-T cell therapy. Tested by Kloss et al., this combination proved capable of eliminating double-positive T cells, suggesting its potential as a new therapeutic strategy for PCa [83].

### Diabodies and bispecific T-cell engagers (BITEs)

Another approach could be to use bispecific antibodies (diabodies) or BITEs [85]. These constructs not only bind to the minimal binding domains (single-chain fragment variables, scFvs) of monoclonal antibodies for CD3ϵ T-cell receptor-associated molecule on the T-cell surface, but also to a specific antigen expressed on the surface of cancer cells. Concurrent engagement of both the specific antigen and CD3 leads to tumor cell lysis through the activation of cytotoxic T-cells, regardless of the TCR-mediated specificity of these cells [86]. The relative specificity and sensitivity of BiTE and CAR constructs has been compared in preclinical models [87]. Given that BiTEs may be beneficial in cancers in which a specific epitope is overexpressed compared with normal tissue, as described by Stone et al., this approach has also been studied in PCa.

Several studies developed and evaluated in vitro the efficacy of these novel antibodies in targeting PSCA and PSMA [83, 88, 89]. However, some failed to block cancer cell proliferation in animal models, only delaying tumor growth, suggesting that diabodies used as a single treatment do not achieve a durable cellular memory response [34]. Despite this, administration of the humanized bispecific antibody MOR209/ES414 in murine xenograft models of human PCa led to the inhibition of tumor growth and increased survival, decreasing PSA expression only in adoptively transferred human T cells [90] A phase I study is ongoing to identify the maximum tolerated dose and to test the clinical activity of ES414 in mPCa patients [NCT02262910].

More recently, AMG 160, a fully human, half-life extended (HLE) BiTE targeting PSMA in PCa cells and CD3 in T cells, demonstrated antitumor activity in xenograft models [91]. Based on these data, a phase I study is underway to evaluate its activity in mPCa patients (NCT03792841). At the 2019 Annual ASCO Meeting, Hummel et al. reported that the PSMA x CD3 BiTE pasotuxizumab demonstrated an acceptable safety profile and dose-dependent clinical activity in mPCa patients [92]. Moreover, theirs was the first study demonstrating BITE clinical activity in solid tumors, 2 long-term responses described in the dose escalation cohort (NCT01723475).

### Epithelial cell adhesion molecules (EpCAM)

EpCAM, also known as CD326, is a stem cell antigen expressed by several solid tumors, including PCa [93, 94]. An EpCAM-CD3 bispecific antibody was recently approved in Europe for patients with malignant ascites. Using this molecule as a TAA, Deng et al. developed EpCAM-specific CARs which not only proved capable of killing PC3M prostate cells (overexpressing EpCAM) but also of prolonging survival in PC3 prostate cells (underexpressing EpCAM). Further investigation is warranted into the role of this molecule in mPCa [84].

## Problems relating to use of CAR-T cell therapy in PCa

The use of CAR-T cells for the treatment of non-hematological tumors exposes the patient to risks that could limit their use in clinical practice. Perhaps the most important risk is the presence of several structures in solid tumors (i.e. extracellular matrix, tumor stroma) that limit the contact between CAR-T cells and the tumor itself [95]. For example, bone is the most frequent site of PCa metastasis. Within this context, the tumor microenvironment enhances aberrant angiogenesis mediated by vascular endothelial growth factor receptor (VEGF) [96]. Shi et al. demonstrated that a combination of immunotherapy and angiogenesis-normalizing treatments increases the efficacy of immunotherapeutic agents [97].

Another issue is the inhibitory tumor microenvironment. Several studies have demonstrated that solid tumors express a higher concentration of programmed death-ligand 1 PD-L1, tryptophan 2,3-dioxygenase, indoleamine 2,3-dioxygenase, IL-10 and regulatory T-cells (Tregs) [98–104]. As Tregs overexpress TGF-β, blocking TGF-β activity could help to improve T cell activity [105]. Kloss et al. evaluated TGF-β overexpression in mice models of aggressive mPCa, reporting enhanced T-cell proliferation, cytokine secretion, in vivo survival and efficacy in destroying cancer cells [77]. As previously reported, bone is the most frequent site of PCa metastasization and different cytokines have been studied for their potential to enable T cells to access bone metastases. In 2000, Kantele et al. used mild radiation treatment or cyclophosphamide chemotherapy to stimulate mPCa cells to express chemokine (C-X-C motif) ligand (CXCL) 12, also known as stromal cell-derived factor (SDF)-1 [106], which is involved in T cell migration to and adhesion on activated endothelium [107].

More recently, some authors evaluated the possibility of inserting a chemokine receptor gene into CAR-T cells. For example, engineering CXCL12 ligand, i.e. C-X-C motif receptor (CXCR)-4, into CAR-T cells could increase the percentage of CAR molecules reaching reach tumor cells [108, 109].

Based on the same hypothesis, other studies have evaluated CAR-T cells engineered to secrete different chemokines, such as CCL2 (involved in tumor homing and vascularization) [110]. Another way of enhancing T cell activity could be to add an immune checkpoint inhibitor to treatment. Combination therapy with CAR-T cells and an anti-PD1 antibody demonstrated higher T-cell activation in a transgenic Her2 mouse model [111]. In PCa, androgen-deprivation therapy combined with T-cells has been evaluated, an in vitro study demonstrating higher cytotoxic activity and proliferation rates of T cells using this treatment strategy. Sanchez et al. showed the feasibility in vitro of a combination of androgen-deprivation therapy and CAR-T cells [112]. Such findings may be attributable to androgen-mediated apoptosis and, consequently, to an increase in TAAs which, in turn, stimulates T-helper activation. For the same reasons, using radiotherapy to induce apoptosis could help to overcome immune inhibition by the tumor microenvironment [34].

However, the solution to CAR-T-related problems is not limited to removing physical or chemical “barriers”. In fact, toxicities caused by the new immunological approach are sometimes difficult to manage. The majority of data on CAR-T-related toxicities originate from hematological trials. Neurological and cardiovascular toxicities, cytokine release syndrome, tumor lysis syndrome and macrophage activation syndrome have all been observed in studies using CD19 CAR-T cells [113–116]. In PCa, the use of prostate-specific antigens could limit systemic immune-related adverse events (IRAEs). Moreover, several molecular options are currently being developed to further reduce the risks of such adverse events. For example, the abovementioned study by Kloss et al. [77] evaluated a combination of low-affinity PSCA-CAR-T cells with high-affinity PSMA-CAR-T cells. Another way of overcoming potential toxicities is to insert an inducible suicide gene into CAR-T cells with the aim of destroying CAR-T cells in the event of serious toxicity. Within this context, Di Stasi et al. demonstrated the role of caspase-9 in inducing T-cell apoptosis [117]. Moreover, some studies suggest that inserting CARs into NK cells or into γδ T-cells could substantially limit the risk of IRAEs [118, 119]. Other recent findings on hematological [120] and solid tumors, including PCa, indicate that the problem might be resolved by developing nanoparticles for CAR-T delivery [28, 121]. However, all of the above issues also limit the use of CAR-T cell therapy in mPCa [122].

## Conclusions

Although TAA-targeting CARs have shown interesting results in pre-clinical studies on mPCa, their clinical use is associated with significant risks for the patient and requires further in-depth investigation. It is therefore essential to draw up toxicity management plans and to identify biomarkers that can predict toxicities such as cytokine-release syndrome. It is still open to debate whether clinical CAR-T cell programs should be managed by bone marrow transplantation teams or by disease-specific teams. This is especially important for solid tumors, where the ideal situation would be to have a team whose expertise comprises bone marrow transplantation in specific diseases.

Numerous issues remain to be resolved, e.g. best TAA to induce safe and effective T-cell activation; best CAR-T cells to use (NK, αβ T cell, γδ T cell); best way to reduce IRAEs in mPCa treated with CAR-T cell treatment. Moreover, is CAR-T cell treatment better than monotherapy, and if not, what is the best combination treatment to improve T-cell activation (CAR-T + antiandrogens; CAR-T + radiotherapy)? Are these combinations safe? Which kind of patient could benefit from CAR-T treatment and which might not? Why? The development of this promising treatment strategy in PCa will depend on these questions being answered, hopefully in the near future.

## Data Availability

Not applicable.

## Abbreviations

ALL: Acute lymphoblastic leukemia
APC: Antigen presenting cells
BITEs: Bispecific T-cell engagers
CAR: Chimeric antigen receptor
CLL: Chronic lymphocytic leukemia
DLBCL: Diffuse large B cell lymphoma
IRAEs: Immune-related adverse events
ITAM: Immune receptor tyrosine-based activation motif
mCRPC: Metastatic castration-resistant PCa
MHC: Major histocompatibility complex
mPCa: Metastatic prostate cancer
NED: Neuroendocrine differentiation
PAP: Prostatic acid phosphatase
PCa: Prostate cancer
PSA: Prostate-specific antigen
PSCA: Prostate stem cell antigen
PSMA: Prostate-specific membrane antigen
scFv: Single-chain fragment variable
SDF: Stromal cell-derived factor
TAA: Tumor-associated antigens
TARP: T-cell receptor gamma alternate reading frame protein
TCR: T cell receptor
VEGF: Vascular endothelial growth factor receptor
